# Möbius bands, unstretchable material sheets and developable surfaces

**DOI:** 10.1098/rspa.2016.0459

**Published:** 2016-08

**Authors:** Yi-chao Chen, Eliot Fried

**Affiliations:** 1 Department of Mechanical Engineering, University of Houston, Houston, TX 77204-4006, USA; 2 Mathematical Soft Matter Unit, Okinawa Institute of Science and Technology Graduate University, Onna, Okinawa 904-0495, Japan

**Keywords:** isometric mappings, inextensible space curves, ruled surfaces, bending elasticity

## Abstract

A Möbius band can be formed by bending a sufficiently long rectangular unstretchable material sheet and joining the two short ends after twisting by 180^°^. This process can be modelled by an isometric mapping from a rectangular region to a developable surface in three-dimensional Euclidean space. Attempts have been made to determine the equilibrium shape of a Möbius band by minimizing the bending energy in the class of mappings from the rectangular region to the collection of developable surfaces. In this work, we show that, although a surface obtained from an isometric mapping of a prescribed planar region must be developable, a mapping from a prescribed planar region to a developable surface is not necessarily isometric. Based on this, we demonstrate that the notion of a rectifying developable cannot be used to describe a pure bending of a rectangular region into a Möbius band or a generic ribbon, as has been erroneously done in many publications. Specifically, our analysis shows that the mapping from a prescribed planar region to a rectifying developable surface is isometric only if that surface is cylindrical with the midline being the generator. Towards providing solutions to this issue, we discuss several alternative modelling strategies that respect the distinction between the physical constraint of unstretchability and the geometrical notion of developability.

## 1. Introduction

In mechanics, an unstretchable material sheet can sustain only deformations corresponding to isometric mappings. Although every surface obtained by bending a flat unstretchable material sheet is developable, a mapping of a prescribed planar region into a developable surface is not necessarily isometric. In spite of this, non-isometric mappings of planar regions into developable surfaces have frequently been used in misguided attempts to describe equilibrium configurations of two-dimensional bodies made from allegedly unstretchable material sheets. The primary purpose of this paper is to clarify the salient issues. Although we focus on Möbius bands, our conclusions apply equally well to orientable twisted strips.

This paper is organized as follows. Some background on the modelling of Möbius bands, as initiated by Sadowsky [1–6], is provided in §2. An expanded discussion of the issues related to the modelling of unstretchable material sheets and the flaws resulting from restricting attention to non-isometric mappings that generate developable surfaces is contained in §3. Basic ideas associated with the parametrization of a surface and the associated notion of a mapping from a planar region to a surface in three-dimensional Euclidean point space are reviewed in §4. The concepts of stretch and curvature, as they relate to the in-plane deformation and out-of-plane bending of a flat material sheet identified with a planar region, are discussed in §5. Developable surfaces and isometric mappings from planar regions to surfaces in space are reviewed in §6. Our main result is established in §7. There we show that, while an isometric mapping always deforms a planar region to a developable surface, a mapping from a planar region to a developable surface need not be isometric. To demonstrate this point, we explicitly work with a class of mappings that have been used frequently to describe deformations of rectangular regions into ribbons and Möbius bands. Any such mapping describes a ruled surface that lies on the rectifying developable of its midline. Most importantly, we establish that the mappings in question are not isometric—except in the trivial case, wherein the mapped surface is cylindrical with the midline being the generator. A concrete illustration of our result is presented in §8. In that illustration, a rectangular region is mapped to a helical ribbon that lies on the surface of a cylinder. In addition to showing that the mapping in question is not isometric, we identify an isometric mapping that takes a parallelogram to the same helical ribbon. The latter mapping is directly relevant to Sadowsky’s [1,2] construction showing that it is possible to bend a rectangular region into a Möbius band without stretching. In §9, we show that a non-isometric mapping from a planar region to a developable surface of the type discussed in §7 can be expressed as the composition of a non-isometric mapping between two planar regions and an isometric mapping to a surface. This is achieved by a straightforward change of independent variables. Finally, a few alternative modelling strategies that respect the distinction between the physical constraint of unstretchability and the geometric notion of developability are discussed in §10.

## 2. Background

In 1930, Sadowsky [1,2] published an appealingly simple constructive proof showing that a rectangular region can be bent, without stretching or tearing, into a Möbius band. In essence, his proof amounts to smoothly joining three helical ribbons bent from parallelograms, with three isosceles trapezoids (two or more of which may degenerate to isosceles triangles), to form a surface with the requisite one-sided spatial connectivity. Recognizing that a strip of paper bent to adopt the shape of his construction would change shape in the absence of externally applied loads, Sadowsky also proposed a variational strategy for determining stable equilibrium shapes of Möbius bands made from unstretchable sheets. That strategy begins by identifying a Möbius band with a developable surface S endowed with bending energy proportional to the integral

2.1 $$\int_{S}H^{2}\;da,$$

where *H* and d*a* denote the mean curvature and element of surface area on S. He stated that ‘the shape of the Möbius band corresponds to a minimum of the bending energy’. For a band of width infinitesimally small compared with its length, Sadowsky claimed that minimizing (2.1) reduces to minimizing an integral

2.2 $$\int_{0}^{l}\kappa^{2}{\left(1+\eta^{2}\right)}^{2}\;ds,$$

over the midline C of S, where *η* is the ratio of the torsion *τ* and the curvature *κ* of C

2.3 $$\eta =\frac{\tau }{\kappa }$$

and d*s* is the element of arclength along C. While he made no attempt to find minimizers of the functional (2.2), Sadowsky [1,2] computed the bending energy for the infinitesimal width-to-length version of his construction, which thus serves as an upper bound for the minimum of the bending energy.

Absent from the discussion leading to (2.2) but present in two contemporaneous papers by Sadowsky [3–6] is the crucial provision that C be locally unstretchable. This postulate is consistent with the absence of the Gaussian curvature *K* of S in (2.1). Sadowsky [3–6] also emphasized that S should lie on the rectifying developable of its midline C. Given an arclength parametrization ***γ*** of C, the rectifying developable of S consists of the envelope of the planes spanned by the tangent and binormal vectors

2.4 $$t=\gamma^{\prime }\;\mathrm{and}\;b=\frac{\gamma^{\prime }\times \gamma^{\prime \prime }}{\kappa }=\frac{t\times t^{\prime }}{\kappa }$$

of the Frenet frame of C. If S lies on the rectifying developable of its midline C of non-vanishing curvature, then it must admit a parametrization determined by an invertible mapping $\hat{\text{ x}}$ from the rectangular region

2.5 D=[0,l]×[−b,b]

of length *l*>0 and half-width *b*>0 to S of the particular form

2.6 $$\hat{\text{x}}(s,t)=\gamma (s)+t(b(s)+\eta (s)t(s)),\;(s,t)\in D.$$

To describe the midline of any smooth closed band, orientable or otherwise, ***γ*** and ***t*** must satisfy closure conditions

2.7 γ(l)=γ(0)andt(l)=t(0).

Moreover, to ensure that a surface S determined in accord with (2.6) is one-sided, there must be an odd number of frame switching points *s*_*i*_∈[0,*l*], namely points *s*_*i*_ at which

2.8 $$\lim_{s↘s_{i}}b(s)=-\lim_{s↗s_{i}}b(s).$$

The assumed invertibility of the mapping $\hat{\text{ x}}$ ensures, without loss of generality, that 1+*tη*^′^(*s*)>0 for each (*s*,*t*) belonging to D. Additionally, the mean curvature *H* and area element *da* of S are given by

2.9 $$H(s,t)=\frac{\kappa (1+\eta^{2}(s))}{2(1+t\eta^{\prime }(s))}\;\mathrm{and}\;da(s,t)=(1+t\eta^{\prime }(s))\;ds\;dt,\;(s,t)\in D.$$

When reducing the problem of minimizing (2.1) to that of minimizing (2.2) for an infinitesimally narrow band, Sadowsky [3,4] referred to an expression *κ*(1+*η*^2^(*s*))/2 for the mean curvature, which is simply the restriction to C of the expression for the mean curvature of S that appears in (2.9).

In what is perhaps the first published paper that recognizes Sadowsky’s contributions to the mechanics of Möbius bands, Wunderlich [7,8] noticed that (2.9) can be used without approximation to yield

2.10 $$\int_{S}H^{2}\;da=\frac{1}{4}\int_{-b}^{b}\int_{0}^{l}\frac{\kappa^{2}{\left(1+\eta^{2}\right)}^{2}}{1+t\eta^{\prime }}\;ds\;dt=\frac{b}{4}\int_{0}^{l}\frac{\kappa^{2}{\left(1+\eta^{2}\right)}^{2}}{b\eta^{\prime }}\log\left(\frac{1+b\eta^{\prime }}{1-b\eta^{\prime }}\right)ds.$$

Subsequent to the papers of Sadowsky [1–6] and Wunderlich [7,8], the practice of restricting attention to surfaces that lie on rectifiable developables has dominated the literature concerning Möbius bands made from unstretchable flat material sheets, as evidenced by earlier studies [9–23]. A remarkable exception to this trend is the comparatively early work of Halpern & Weaver [24], who used homotopy methods to prove that a flat rectangular strip of half-width *b* and length *l* admits an isometric immersion as a Möbius band in three-dimensional Euclidean point space if and only if *πb*<*l* and, moreover, conjectured that such a strip can be isometrically embedded as a Möbius band in three-dimensional Euclidean point space only if the more restrictive inequality $2\sqrt{3}b<l$ holds. It is noteworthy that Sadowsky [1,2] imposed the latter inequality to ensure the viability of his construction.

## 3. Critique

It appears to have gone unnoticed that an unstretchable flat rectangular sheet identified with a region D=[0,l]×[−b,b] *cannot* in general sustain deformations described by mappings of the form (2.6). In §6 of the present paper, we show that a mapping $\hat{\text{ x}}$ defined in (2.6) is not isometric unless *τ*=0 at each point along the midline C, which must therefore be planar. Any claim that a parametrization of the form (2.6) generally provides an isometric embedding, into three-dimensional Euclidean point space, of a rectangular region is consequently false. It is important to recognize that, if *τ*=0 on a closed curve C, the unit binormal ***b*** of the Frenet frame of C must be perpendicular to the plane in which C resides and C must have an even number of frame switching points. This being so, (2.6) cannot parametrize a Möbius band.

The oversight described above might stem from a misinterpretation of the commonly encountered characterization of a developable surface, which states that any such surface can be mapped isometrically to a planar region. Any surface parametrized in accordance with (2.6) is indeed developable and can, therefore, be mapped isometrically to a planar region. However, setting aside the degenerate case where *τ* vanishes at each point along the midline C, the isometric flattening of the surface S parametrized by a mapping $\hat{\text{ x}}$ from D to S of the form defined in (2.6) need not be D.

This error inevitably undermines any effort to determine stable equilibrium shapes of Möbius bands by minimizing Wunderlich’s functional (2.10). As the isometric flattenings of two surfaces parametrized by different mappings of the form (2.6) for a given region D generally differ, comparing the corresponding values of the bending energy (2.10) amounts not to comparing the energies of two possible isometric bendings of an unstretchable rectangular region of half-width *b* and length *l* but rather to juxtaposing the energies of two differently shaped flat regions mapped into developable surfaces. Hence, such a comparison cannot generally yield useful information towards determining the equilibrium shape of the Möbius band made from an unstretchable sheet of given prescribed shape. Moreover, as using $\hat{\text{ x}}$ defined in (2.6) to map D to a surface S must inevitably involve in-plane stretching, the influence of the associated stretching energy on shape would generally be non-negligible. Unfortunately, that influence has been neglected in all computations based on minimizing only the bending energy (2.10).

Our conclusions regarding the family of mappings defined in (2.6) are independent of whether the condition (2.8) needed to ensure that $\hat{\text{ x}}$ describes a Möbius band is satisfied. Specifically, such mappings cannot generally be used to reliably determine the shape of a surface made by bending an unstretchable rectangular sheet. Quantifying the magnitude of the error incurred by using (2.6) to approximate an isometric mapping of a narrow ribbon remains an interesting question. A satisfactory answer to this question would provide useful guidelines for numerical strategies based on minimizing the bending energy functional (2.10), such as those used by Starostin & van der Heijden [22] and Shen *et al.* [23]. Even so, there is no reason to believe, *a priori*, that the class of mappings (2.6) is rich enough to include all possible equilibrium shapes of such a sheet.

## 4. Parametrization of a surface: deformations and associated gradients

Consider a surface S, in three-dimensional Euclidean point space, with parametric representation

4.1 $$x=\tilde{x}(s,t),$$

where ***x*** denotes a generic point on S and where *s* and *t* are parameters. A parametrization of a surface is not unique and need not be tied to a physical process. If, however, the surface represents a configuration occupied by a material sheet, then the situation changes markedly. Under such circumstances, each ordered pair (*s*,*t*) serves to label a unique material particle of the sheet in some (possibly flat) reference configuration and $\tilde{x}$ encodes the changes in particle positions needed to surjectively map the sheet from that configuration onto the surface S. This is the view taken in many works, the present one included. Among other things, adopting this view makes it possible to apply classical kinematical notions from mechanics to describe changes in the geometry of a surface.

In mechanics, the mapping $\tilde{x}$ in (4.1) is an example of a deformation—a physical process that, for example, changes the shape of a two-dimensional rectangular region into a Möbius band. We adopt a more compact notation by using a vector ***r*** to denote the independent variable on which the mapping $\tilde{x}$ depends, with the parameters *s* and *t* being interpreted as the components of ***r*** with respect to an orthonormal basis. This appears to be consistent with the formulation of many authors who treat the parameters *s* and *t* as the coordinates of a point in the planar region relative to an orthogonal Cartesian coordinate system. We denote the vectorial translation spaces associated with $E^{2}$ or $E^{3}$ by $V^{2}$ or $V^{3}$, respectively.

Introducing a planar region R, which we identify with a flat material sheet, in $E^{2}$, we thus replace (4.1) by

4.2 $$x=\tilde{x}(r),\;r\in R.$$

The gradient of $\tilde{x}$ on R, denoted by ***F***, is called the deformation gradient. Each value of ***F*** is a linear transformation that maps $V^{2}$ into $V^{3}$. We assume that the restriction of $F:E^{2}\to E^{3}$ to its range is locally invertible. To describe the notion of curvature, it is helpful to introduce the second-order deformation gradient ***G***, which can be viewed as a linear transformation from $V^{2}\times V^{2}$ into $V^{3}$. For brevity, we refer to ***G*** as the ‘second gradient’.

## 5. Stretch and curvature

The stretch associated with a deformation $\tilde{x}$ of a planar region R to a surface S and the curvature of S can be described in terms of the deformation gradient ***F*** and the second gradient ***G***.

### (a) Stretch

We employ the usual Euclidean norms and the corresponding inner products in the domain R and codomain $E^{3}$ of the mapping $\tilde{x}$. Of central kinematical importance is the polar decomposition

5.1 F=RU

of ***F***, where ***R*** is a linear transformation from $V^{2}$ to $V^{3}$ and ***U*** is a linear transformation (called the stretch tensor) from $V^{2}$ to $V^{2}$. For completeness, we provide a derivation of (5.1).

The Cauchy–Green deformation tensor ***C*** is defined by

5.2 $$C={\text{F}}^{⊤}F,$$

where ${\text{ F}}^{⊤}:V^{3}\to V^{2}$ is defined in the usual way through inner products involving the domain and codomain of ***F***. It follows from (5.2) that $C:V^{2}\to V^{2}$ is symmetric. By the assumption that $\tilde{x}$ is locally invertible, ***C*** is positive-definite and therefore has a square root $U:V^{2}\to V^{2}$, which satisfies

5.3 $$U^{2}=C$$

and is itself symmetric and positive-definite. In mechanics, ***U*** is called the stretch tensor. We now define tensor $R:V^{2}\to V^{3}$ by

5.4 $$R=FU^{-1}.$$

For arbitrary elements ***a*** and ***b*** of $E^{2}$, we may use (5.2)–(5.4) to find that

5.5 (Ra)⋅(Rb)=a⋅b,

where the inner products on the left- and right-hand sides of the equation are those defined on $V^{3}$ and $V^{2}$, respectively. Using conventional arguments, we find from (5.5) that ***R*** preserves the length of any line segment in R and the angle between any two line segments in R. In this sense, ***R*** can be thought of as a linear transformation that rotates vectors in $V^{2}$ into vectors in $V^{3}$.

A mapping $\tilde{x}$ from a planar region R in $E^{2}$ to a surface S in $E^{3}$ is isometric if it preserves the length of an arbitrary curve $\left\{r=\tilde{r}(u),u_{0}\leq u\leq u_{1}\right\}$ traced on R while mapping the curve into a space curve on S,

5.6 $$\int_{u_{0}}^{u_{1}}\left|\frac{d\tilde{x}(\tilde{r}(u))}{du}\right|du=\int_{u_{0}}^{u_{1}}\left|\frac{d\tilde{r}(u)}{du}\right|du.$$

Using the particular choice

5.7 $$\tilde{r}(u)=r_{0}+(u-u_{0})a$$

shows that (5.6) holds only if

5.8 |Fa|=|a|

for each ***a*** in $V^{2}$, or, equivalently,

5.9 C=I.

Equations (5.8) and (5.9) are also sufficient for $\tilde{x}$ to be isometric. Thus, for $\tilde{x}$ to be an isometric mapping, the associated Cauchy–Green deformation tensor ***C***, and hence the stretch tensor ***U***, must be the identity tensor. The material sheet identified with R is said to be unstretchable if it is capable of sustaining only deformations for which ***C***=***I*** or, equivalently, ***U***=***I***.

### (b) Curvature

Given linearly independent elements ***c*** and ***d*** of $V^{2}$, a unit normal vector to S is determined by

5.10 $$n=\frac{Fc\times Fd}{|Fc\times Fd|}.$$

The second fundamental form of the surface S can be represented by a tensor $D:V^{2}\to V^{2}$ given in terms of ***n*** and ***G*** by

5.11 D=nG,

where ***nG*** is defined so that ***a***⋅((***nG***)***b***)=***n***⋅***G***[***a***⊗***b***]=***G***⋅(***n***⊗***a***⊗***b***) for all choices of ***a*** and ***b*** in $E^{2}$.

The curvatures of the surface S can be expressed in terms of the first and second fundamental forms. For completeness, a brief derivation of the relevant expressions is provided next. Given an element ***a*** of $V^{2}$, the normal curvature *κ* of the surface S in the direction of ***Fa*** is defined by

5.12 $$\kappa =\frac{a\cdot Da}{a\cdot Ca}.$$

The principal curvatures *κ*_1_ and *κ*_2_ of S correspond to the minimum and maximum values of *κ* for all ***a*** in $V^{2}$, and can be found by solving the equation *dκ*/*d****a***=**0**, which leads to

5.13 Da=κCa,

or, by the local invertibility of $\tilde{x}$,

5.14 $$C^{-1}Da=\kappa a,$$

from which it follows that *κ*_1_ and *κ*_2_ are the eigenvalues of ***C***^−1^***D***. Additionally, the mean and Gaussian curvatures *H* and *K* of S are defined in terms of *κ*_1_ and *κ*_2_ by

5.15 $$H=\frac{1}{2}(\kappa_{1}+\kappa_{2})=\frac{1}{2}\;\mathrm{tr}(C^{-1}D)=\frac{1}{2}C^{-1}\cdot D$$

and

5.16 $$K=\kappa_{1}\kappa_{2}=\det(C^{-1}D)=\frac{\det D}{\det C}.$$

Moreover, the curvature tensor (or Weingarten map) $L=-{\mathrm{grad}}_{S}n$ of S is related to ***D*** by

5.17 $${\text{F}}^{⊤}LF=D.$$

Finally, substituting (5.17) into (5.13), using (5.2), and defining an element ***v*** of $V^{3}$ by ***v***=***Fa***, we find that

5.18 Lv=κv,

from which it follows that *κ*_1_ and *κ*_2_ are also the eigenvalues of ***L***.

## 6. Developable surfaces and isometric mappings

A surface is said to be developable if its Gaussian curvature vanishes everywhere. It is common knowledge that the image of a planar region under an isometric mapping must be a developable surface. As a basis for the ensuing discussion, we provide a simple proof of this fact.

Let $\tilde{x}$ be an isometric mapping defined on some planar region R. Then, by (5.2) and (5.9), the associated deformation gradient ***F*** must satisfy

6.1 $${\text{F}}^{⊤}F=I.$$

Hence, ***F*** must satisfy

6.2 Fa⋅Fb=a⋅b

for all ***a*** and ***b*** in $V^{2}$. On taking the gradient of (6.2), we find that

6.3 Fa⋅G[b⊗c]+Fb⋅G[a⊗c]=0

for all ***a***, ***b*** and ***c*** in $V^{2}$, from which we obtain the conditions

6.4 Fa⋅G[a⊗a]=0,Fa⋅G[a⊗b]=0andFa⋅G[b⊗b]=−Fb⋅G[a⊗b]=0.

Equivalently, (6.4) can be written as a single relation

6.5 Fa⋅G[A]=0

for all ***a*** in $V^{2}$ and all ***A*** in the space $\mathrm{Lin}(V^{2})$ of all linear transformations on $V^{2}$. Next, choosing orthonormal elements ***a*** and ***b*** of $E^{2}$ (so that |***a***|=|***b***|=1 and ***a***⋅***b***=0) and defining elements **ℓ** and ***m*** of $V^{3}$ by **ℓ**=***Fa*** and ***m***=***Fb***, we find, as a consequence of (5.11) and (6.5), that the second gradient admits a representation of the form

6.6 G=(ℓ⊗ℓ+m⊗m+n⊗n)G=n⊗nG=n⊗D.

Next, taking the gradient of (6.3), we find that

6.7 f(a,b,c,d)=0

for all ***a***, ***b***, ***c*** and ***d*** in $V^{2}$, where $f:V^{2}\times V^{2}\times V^{2}\times V^{2}\to R$ is given by

6.8 $$\begin{array}{rl} f(a,b,c,d) & =G[a⊗d]\cdot G[b⊗c]+G[b⊗d]\cdot G[a⊗c]\\ & \;+Fa\cdot H[b⊗c⊗d]+Fb\cdot H[a⊗c⊗d], \end{array}$$

with ***H*** being the third-order deformation gradient. Taking advantage of the various symmetries of ***G*** and ***H***, we find from (6.8) that

6.9 $$\begin{array}{rl} & f(a,a,b,b)-\frac{1}{2}(\;f(a,b,a,b)+f(b,a,b,a))\\ & \;=G[a⊗a]\cdot G[b⊗b]-\frac{1}{2}(G[a⊗b]\cdot G[a⊗b]+G[b⊗a]\cdot G[b⊗a])=0. \end{array}$$

Next, using the representation (6.6) for ***G*** in (6.9), we obtain

6.10 $$(a\cdot Da)(b\cdot Db)-{\left(a\cdot Db\right)}^{2}=0.$$

Finally, recalling that ***a*** and ***b*** are orthonormal, we recognize the left-hand side of (6.10) as the determinant of ***D*** and, with reference to definition (5.16) of the Gaussian curvature, we conclude that

6.11 $$K=\frac{\det D}{\det C}=0.$$

We have shown that if a mapping $\tilde{x}$ from a given planar region R in $E^{2}$ to a surface S in $E^{3}$ is isometric, then S must be developable. However, the converse is not true. It is possible to map a planar region R to a developable surface S non-isometrically. For instance, the entire plane can be mapped into itself non-isometrically. A less trivial example, which we will next explore in detail, is provided by the particular class of mappings $\hat{\text{ x}}$ defined in (2.6), which has prevailed in the literature in the efforts to model ribbons and bands. Our analysis also leads unambiguously to the conclusion that the mappings belonging to this class are *not* generally isometric, and, therefore, are unsuitable for modelling unstretchable material sheets.

## 7. Mappings from planar regions to rectifying developable surfaces are typically not isometric

Consider a surface S parametrized by a mapping $\hat{\text{ x}}$ of the form (2.6), which is recast in the current notation as

7.1 $$\hat{\text{x}}(r)=\gamma (r_{1})+r_{2}(b(r_{1})+\eta (r_{1})t(r_{1})),\;r\in D,$$

where *r*_1_=***r***⋅***e***_1_ and *r*_2_=***r***⋅***e***_2_ are the components of ***r*** with respect to a positively oriented orthonormal basis {***e***_1_,***e***_2_} for $V^{2}$,

7.2 $$D=\{r\in E^{2}:0\leq r_{1}\leq l,|r_{2}|\leq b\}$$

is a rectangular region in $E^{2}$, ***γ*** represents a unit speed curve C in $E^{3}$,

7.3 $$t=\gamma^{\prime },\;p=\frac{t^{\prime }}{\kappa }\;\mathrm{and}\;b=t\times p$$

are the tangent, normal and binormal vectors of C, respectively, *κ*=|***t***^′^| and *τ*=***t***⋅(***p***×***p***^′^) are the curvature and torsion of C, respectively, and *η* is the ratio

7.4 $$\eta =\frac{\tau }{\kappa }.$$

The deformation gradient ***F*** of $\hat{\text{ x}}$ is

7.5 $$\begin{array}{rl} F & =\gamma^{\prime }⊗e_{1}+r_{2}(b^{\prime }+\eta^{\prime }t+\eta t^{\prime })⊗e_{1}+(b+\eta t)⊗e_{2}\\ & =(1+r_{2}\eta^{\prime })t⊗e_{1}+(b+\eta t)⊗e_{2}, \end{array}$$

where we have made use of (7.4) and the Frenet–Serret relations ***t***^′^=*κ****p***, ***p***^′^=−*κ****t***+*τ****b*** and ***b***^′^=−*τ****p***. Importantly, by invoking (7.4), we have tacitly assumed that the curvature *κ* of C is non-vanishing. In this regard, it is worth observing that if the curvature *κ* of C vanishes in an interval of [0,*l*], then the unit normal ***p*** and unit binormal ***b*** of the Frenet frame C are undefined on that interval. Under these circumstances, the notion of a rectifying developable, and thus a mapping of the form (7.1), becomes completely irrelevant.

The second gradient ***G*** of $\hat{\text{ x}}$ is given by

7.6 $$\text{G}=(\kappa p+r_{2}(\eta^{\prime \prime }t+\kappa \eta^{\prime }p))⊗e_{1}⊗e_{1}+\eta^{\prime }t⊗(e_{1}⊗e_{2}+e_{2}⊗e_{1}).$$

Further, the normal vector ***n*** to the surface S parametrized by $\hat{\text{ x}}$ can be found from (5.10), with ***c*** and ***d*** being replaced by ***e***_1_ and ***e***_2_, respectively. Assuming, consistent with the previously stipulated invertibility of $\hat{\text{ x}}$, that 1+*r*_2_*η*^′^(*r*_1_)>0 for all ***r*** in D, this yields

7.7 $$n=\frac{Fe_{1}\times Fe_{2}}{|Fe_{1}\times Fe_{2}|}=\frac{\left(1+r_{2}\eta^{\prime })t\times (b+\eta t\right)}{|(1+r_{2}\eta^{\prime })t\times (b+\eta t)|}=-p,$$

whereby the unit normal of S is directed opposite to the unit normal of the Frenet frame of the midline C. As a consequence of (7.6) and (7.7), it follows that

7.8 $$D=n\text{G}=\kappa (1+r_{2}\eta^{\prime })e_{1}⊗e_{1}.$$

Thus, det(n G) vanishes and we infer from (5.16) that the corresponding Gaussian curvature vanishes,

7.9 $$K=\frac{\det D}{\det C}=0.$$

A mapping $\hat{\text{ x}}$ of the form defined in (7.1) therefore deforms the rectangular region D to a developable surface S.

Although the image of a mapping $\hat{\text{ x}}$ of the form (7.1) is a developable surface, the mapping $\hat{x}$ is *not* isometric unless it satisfies some highly restrictive conditions that rule out the intended utility of (7.1). To verify the foregoing assertion, we first use (7.5) in (5.2) to give

7.10 $$C=F^{⊤}F={\left(1+r_{2}\eta^{\prime }\right)}^{2}e_{1}⊗e_{1}+\eta (1+r_{2}\eta^{\prime })(e_{1}⊗e_{2}+e_{2}⊗e_{1})+(1+\eta^{2})e_{2}⊗e_{2}.$$

Inspection of (7.10) then reveals that the Cauchy–Green tensor ***C*** corresponding to a mapping $\hat{\text{ x}}$ of the form (7.1) coincides with the two-dimensional identity tensor ***I*** if and only if *η* vanishes at each point of the midline C of the surface S parametrized by $\hat{\text{ x}}$ or, with reference to the definition (2.3) of *η*, if and only if the torsion *τ* vanishes at each point of C with non-vanishing curvature *κ*.

If *κ* and *τ* obey *κ*>0 and *τ*=0 at each point of C, then C must be planar and the unit binormal ***b*** of the Frenet frame of C must be perpendicular to the plane in which C resides. In other words, the rectangular planar region D must be mapped to a cylindrical surface with the midline C being perpendicular to the axis of the cylinder. Only in this degenerate case is the mapping $\hat{\text{ x}}$ defined in (7.1) isometric. Importantly, as condition (2.8) for frame switching points cannot be met in this special case, $\hat{\text{ x}}$ can never be used to describe a pure bending of a planar region into a Möbius band.

It is noteworthy that the requirement necessary to ensure that a mapping $\hat{\text{ x}}$ of the form (2.6) is isometric, namely that *τ* vanishes identically, is also necessary to preserve the lengths of the lines of constant *r*_1_ in the region D and, thus, to ensure that the isometric flattening of the ruled surface S parametrized by $\hat{\text{ x}}$ is rectangular. To verify this assertion, it suffices to notice that the rule vector ***b***+*η****t*** is of magnitude $\sqrt{1+\eta^{2}}$. Thus, if *η*(*s*)≠0 at some *r*_1_ in [0,*l*], then the corresponding line in the planar region determined by the isometric flattening of S is of length $2\sqrt{1+\eta^{2}(r_{1})}b>2b$. It follows that $\hat{\text{ x}}$ elongates material filaments along ***e***_2_ unless *τ*≡0.

Randrup & Røgen [12] and Sabitov [14] considered an alternative to the parametrization (7.1) for a surface S on the rectifying developable of its midline C. Their alternative involves a mapping $\bar{x}$ of the form

7.11 $$\bar{x}(r)=\gamma (r_{1})+\frac{r_{2}(b(r_{1})+\eta (r_{1})t(r_{1}))}{\sqrt{1+\eta^{2}(r_{1})}},$$

which, in contrast to a mapping $\hat{\text{ x}}$ of the form (2.6), would preserve the lengths of the lines of constant *r*_1_ in the rectangular region D defined in (7.2). Despite this, calculations entirely analogous to those leading to (7.10) show that the Cauchy–Green tensor associated with $\bar{x}$ coincides with the identity tensor if and only if *η*=0 (or equivalently *τ*=0) at each point of C. Generally, a surface that lies on the rectifying developable of its midline has the parametrization of the form

7.12 $$\overset{ˇ}{x}(r)=\gamma (r_{1})+r_{2}(\alpha (r_{1})b(r_{1})+\beta (r_{1})t(r_{1})),$$

of which (7.1) and (7.11) are special cases. It is readily shown that such a surface is developable if and only if *β*(*ατ*−*βκ*)=0, and that mapping (7.12) is isometric if and only if *α*=1,*β*=0 and ***b*** is constant. This is in complete agreement with the conclusion drawn above.

In an effort to establish the existence of an embedding of a Möbius band in three-dimensional Euclidean point space, Chicone & Kalton [25] considered a class of developable surfaces with parametrization

7.13 $$\overset{`}{x}(r)=\gamma (r_{1})+r_{2}\omega (r_{1}),$$

where ***ω*** is a unit vector-valued function. The parametrization (7.13) encompasses (7.12), and therefore (7.1) and (7.11), as special cases. It is easy to show that the parametrization (7.13) is isometric if and only if ***ω*** is constant and everywhere orthogonal to ***t***=***γ***^′^. This is again in agreement with the central conclusion of the present work.

Because the calculations performed in this section rely on representing vector and tensor fields in terms of their components relative to a fixed rectangular Cartesian basis, it is perhaps natural to wonder whether the results remain true if a curvilinear basis is used instead. Granted that the rectangular region D is also an admissible configuration, which seems to be an entirely reasonable assumption, Chen & Fried [26] demonstrate that the results of this section hold independent of basis.

## 8. A relevant example

### (a) General helical ribbons

We now provide an explicit example of a non-isometric mapping of the form (7.1) that takes the rectangular region $D=\{r\in E^{2}:0\leq r_{1}\leq l,|r_{2}|\leq b\}$ to a developable surface S. We further show that the surface S can be mapped isometrically only onto a planar region $\tilde{D}$ that is not rectangular. The latter mapping is directly relevant to the curved portions of Sadowsky’s [1,2] construction of a Möbius band. For simplicity, we choose and fix a positively oriented orthonormal basis {***e***_1_,***e***_2_,***e***_3_} for $V^{3}$ and represent all vectors and tensors in terms of their components relative to that basis. We emphasize that ***e***_1_ and ***e***_2_ need not be the base vectors previously used for $V^{2}$. Our example is based on a circular helix H with axis ***e***_3_, radius *ρ*, pitch angle *θ*, and arclength *l*>0. Invoking the well-known identities

8.1 $$\kappa =\frac{\cos^{2}\theta }{\rho }>0\;\mathrm{and}\;\tau =\frac{\sin\theta \cos\theta }{\rho }>0$$

for the curvature and torsion of such a helix and recalling definition (2.3) of *η*, we find that

8.2 $$\eta =\frac{\tau }{\kappa }=\tan\theta >0.$$

The helix H thus admits an arclength parametrization of the form

8.3 $$\gamma (r_{1})=o+\rho \left(\cos\frac{(\cos\theta )r_{1}}{\rho }e_{1}+\sin\frac{(\cos\theta )r_{1}}{\rho }e_{2}\right)+(\sin\theta )r_{1}\;e_{3},\;r_{1}\in [0,l],$$

where ***o*** denotes the point at the origin. Moreover, using (2.4), (8.2) and (8.3), we find that the combination ***b***+*η****t*** is constant and given by

8.4 $$b+\eta t=(\sec\theta )e_{3}.$$

With reference to (7.1), we now combine (8.3) and (8.4) to yield a parametrization

8.5 $$\begin{array}{rl} \hat{\text{x}}(r) & =\gamma (r_{1})+r_{2}(b(r_{1})+\eta t(r_{1}))\\ & =o+\rho \left(\cos\frac{(\cos\theta )r_{1}}{\rho }e_{1}+\sin\frac{(\cos\theta )r_{1}}{\rho }e_{2}\right)+((\sin\theta )r_{1}+(\sec\theta )r_{2})e_{3},\;r\in D, \end{array}$$

of a family of ruled surfaces that lie on the rectifying developable of H. To ensure that this parametrization is free of overlap for arbitrary *l*>0, we require in (8.5) that

8.6 b≤πρsin⁡θ.

The surface S described by (8.5) is a helical ribbon that lies on the surface of a cylinder with axis parallel to ***e***_3_ and radius *ρ*.

Since a mapping $\hat{\text{ x}}$ of the form (7.1) is isometric if and only if *τ* (and, thus, *η*) vanishes, we conclude from (8.2) that the particular mapping $\hat{\text{ x}}$ defined in (8.5) is *not* isometric. Indeed, by (7.10) and (8.2), the Cauchy–Green tensor ***C*** corresponding to (8.5) has the representation

8.7 $$C=e_{1}⊗e_{1}+\tan\theta (e_{1}⊗e_{2}+e_{2}⊗e_{1})+(\sec^{2}\theta )e_{2}⊗e_{2}$$

and thus does not coincide with the two-dimensional identity tensor ***I*** for any non-zero pitch angle *θ*.

The mapping $\hat{\text{ x}}$ defined in (8.5) takes the rectangular region D to a surface S which coincides with a portion of the surface of a cylinder and is clearly developable. It therefore follows that S can be mapped isometrically to a planar region $\tilde{D}$. Although the developable surface S cannot be obtained by an isometric mapping from the rectangle D, we show below that it can be obtained by an isometric mapping $\tilde{x}$ from a planar region $\tilde{D}$ which is *not* rectangular.

The planar region $\tilde{D}$ and the isometric mapping $\tilde{x}$ can be conveniently found on making reference to the polar decomposition (5.1), which can be interpreted as a composition involving the gradients of two mappings associated with $\hat{\text{ x}}$ defined in (8.5). The first element ***U*** in the composition is the gradient of a mapping that non-isometrically deforms *D* to another planar region, which is identified as $\tilde{D}$. The second element ***R*** in the composition is the gradient of a mapping that isometrically deforms the planar region $\tilde{D}$ to the surface S. The inverse ***R***^⊤^ of the second element ***R*** of the composition is also isometric and maps S to $\tilde{D}$. The specific form of ***U*** corresponding to the mapping $\hat{\text{ x}}$ defined in (8.5) can be obtained by computing the square root of the Cauchy–Green tensor ***C*** in (8.7) and has the representation

8.8 $$U=\frac{2e_{1}⊗e_{1}+\tan\theta (e_{1}⊗e_{2}+e_{2}⊗e_{1})+(1+\sec^{2}\theta )e_{2}⊗e_{2}}{\sqrt{3+\sec^{2}\theta }}.$$

This stretch tensor takes the rectangular region D to a parallelogram which can be rotated so that its midline coincides with that of D. The corresponding rotation tensor is found to be

8.9 $$Q=\frac{2e_{1}⊗e_{1}+\tan\theta (e_{1}⊗e_{2}-e_{2}⊗e_{1})+2e_{2}⊗e_{2}}{\sqrt{3+\sec^{2}\theta }}.$$

It then follows that the tensor

8.10 $$QU=e_{1}⊗e_{1}+\tan\theta \;e_{1}⊗e_{2}+e_{2}⊗e_{2}$$

takes D to a parallelogram, which we identify as $\tilde{D}$. If the rectangular region D is given (7.2), the region $\tilde{D}$ is the parallelogram defined by

8.11 $$\tilde{D}=\{r\in E^{2}:0\leq r_{1}-(\tan\theta )r_{2}\leq l,|r_{2}|\leq b\}.$$

The (*r*_1_,*r*_2_)-coordinates of the vertices of $\tilde{D}$ are (−btan⁡θ,−b), (l−btan⁡θ,−b), (l+btan⁡θ,b) and (btan⁡θ,b). The isometric flattening of the surface S arising from (8.5) is therefore the parallelogram $\tilde{D}$ instead of the domain D of the mapping $\hat{\text{ x}}$ defined in (8.5). Consistent with the contents of the paragraph immediately prior to the paragraph containing (7.11), the images of the lines of constant *r*_1_ in the isometric flattening of S are straight lines of length $2\sqrt{1+\tan^{2}\theta }b>2b$.

Moreover, the tensor (***QU***)^−1^ takes $\tilde{D}$ back to D. The isometric mapping $\tilde{x}$ that takes $\tilde{D}$ to S is therefore given by

8.12 $$\begin{array}{rl} \tilde{x}(r) & =\hat{\text{x}}({\left(QU\right)}^{-1}r)\\ & =o+\rho \left(\cos\frac{(\cos\theta )r_{1}-(\sin\theta )r_{2}}{\rho }e_{1}+\sin\frac{(\cos\theta )r_{1}-(\sin\theta )r_{2}}{\rho }e_{2}\right)\\ & \;+((\sin\theta )r_{1}+(\cos\theta )r_{2})e_{3}. \end{array}$$

To confirm that the mapping $\tilde{x}$ defined by (8.12) is isometric, it suffices to calculate the corresponding Cauchy–Green tensor ***C***. The differences between the expression for $\hat{\text{ x}}$ in (8.5) and (8.12) are both obvious and non-trivial. Most conspicuously, although the mapping $\hat{\text{ x}}$ defined by (8.5) is of the form (7.1), the mapping $\tilde{\text{ x}}$ defined in (8.12) cannot be put into the form (7.1). This example demonstrates unambiguously that a mapping $\hat{\text{ x}}$ of the form (7.1) can parametrize a developable surface that is *not* an isometric mapping of the rectangular region D and, thus, provides a concrete illustration of our general result concerning all such mappings.

### (b) Specific helical ribbon

For illustrative purposes, we take

8.13 $$\rho =\frac{l}{\sqrt{2}},\;b=\frac{l}{\pi }\;\mathrm{and}\;\theta =\frac{\pi }{4}.$$

Granted these choices, (8.1) and (8.2) yield

8.14 $$\kappa =\tau =\frac{1}{\sqrt{2}\rho }\;\mathrm{and}\;\eta =1.$$

Moreover, since $\pi \rho \sin\theta =\pi^{2}b/2>b$, these choices comply with the restriction (8.6) needed to ensure that the parametrization is free of overlap. For simplicity and without loss of generality, we take the half-width *b* to be of unit length, in which case it follows that

8.15 $$\hat{\text{x}}(r)=o+\frac{\cos r_{1}\;e_{1}+\sin r_{1}\;e_{2}+(r_{1}+2r_{2})e_{3}}{\sqrt{2}},\;r\in D.$$

**Figure 1.**
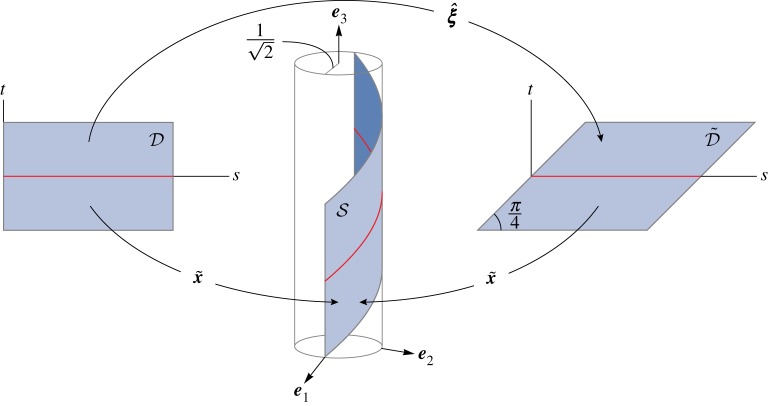
A mapping from a planar region to a developable surface need not be isometric and, thus, is generally inconsistent with the constraint of material unstretchability: a mapping $\hat{\text{ x}}$ of the general form (7.1), with the specific form (8.15), takes a rectangular region $D=\{r\in E^{2}:0\leq r_{1}\leq l,|r_{2}|\leq b\}$ to a helical ribbon S that lies on a cylinder of radius $1/\sqrt{2}$ and axis directed along ***e***_3_. The midline of S, indicated in red, is a circular helix with axis ***e***_3_, radius $\rho =1/\sqrt{2}$, pitch angle *θ*=*π*/4 and length *π*. Its preimages in D and $\tilde{D}$, also indicated in red, are also of length *π*. Whereas S is not isometric to D, it is isometric to the parallelogram $\tilde{D}=\{r:0\leq r_{1}-r_{2}\leq \pi ,|r_{2}|\leq 1\}$ with acute interior angle *π*/4, horizontal side length *π* and inclined side length $2\sqrt{2}$. The mapping $\tilde{x}$ defined in (8.20) is, on the contrary, isometric and accordingly bends $\tilde{D}$ into S without stretching. The mapping $\hat{\xi }$ that takes D onto $\tilde{D}$ describes a homogeneous simple shear. (To avoid clutter, the basis vectors ***e***_1_, ***e***_2_ and ***e***_3_ are placed with their tails emanating from the points with coordinates $\left(1/\sqrt{2},0,-1/\sqrt{2}\right)$, $\left(0,1/\sqrt{2},-1/\sqrt{2}\right)$ and $\left(0,0,(\pi +2)/\sqrt{2}\right)$ relative to the Cartesian coordinate system with origin ***o*** and basis {***e***_1_,***e***_2_,***e***_3_}.)

The mapping $\hat{\text{ x}}$ defined in (8.15) maps the rectangular region D to a surface S which is a portion of the surface of a cylinder of radius $1/\sqrt{2}$, as depicted in figure 1. In particular, relative to the Cartesian coordinate system with origin ***o*** and basis {***e***_1_,***e***_2_,***e***_3_}, it, respectively, maps the corners (0,−1), (*π*,−1), (*π*,1) and (0,1) of D to the points with coordinates

8.16 $$\left(\frac{1}{\sqrt{2}},0,-\frac{2}{\sqrt{2}}\right),\;\left(-\frac{1}{\sqrt{2}},0,\frac{\pi -2}{\sqrt{2}}\right),\;\left(-\frac{1}{\sqrt{2}},0,\frac{\pi +2}{\sqrt{2}}\right)\;\mathrm{and}\;\left(\frac{1}{\sqrt{2}},0,\frac{1}{\sqrt{2}}\right).$$

The transformation tensor ***QU*** defined in (8.10) specializes to

8.17 $$QU=e_{1}⊗e_{1}+e_{1}⊗e_{2}+e_{2}⊗e_{2}.$$

We define a mapping $\hat{\xi }:D\to E^{2}$ by

8.18 $$\hat{\xi }(r)=QUr=(r_{1}+r_{2})e_{1}+r_{2}e_{2}.$$

This mapping is a homogeneous simple shear that takes the rectangle D onto the parallelogram

8.19 $$\tilde{D}=\{r\in E^{2}:0\leq r_{1}-r_{2}\leq \pi ,|r_{2}|\leq 1\}.$$

Crucially, whereas the rectangle D cannot be mapped isometrically to S, the parallelogram $\tilde{D}$ can be mapped isometrically to S, as depicted in figure 1. The (*r*_1_,*r*_2_)-coordinates of the vertices of $\tilde{D}$ are (−1,−1), (*π*−1,−1), (*π*+1,1) and (1,1). For the particular example (8.15), material fibres oriented along ***e***_2_ are elongated by a factor of $\sqrt{2}$.

Moreover, the isometric mapping $\tilde{x}$ defined in (8.12) specializes to

8.20 $$\tilde{x}(r)=o+\frac{\cos(r_{1}-r_{2})e_{1}+\sin(r_{1}-r_{2})e_{2}+(r_{1}+r_{2})e_{3}}{\sqrt{2}}.$$

It is readily verified that the isometric mapping (8.20) takes the parallelogram $\tilde{D}$ to S. In particular, relative to the Cartesian coordinate system with origin ***o*** and basis {***e***_1_,***e***_2_,***e***_3_}, $\tilde{x}$ maps the vertices of (−1,−1), (*π*−1,−1), (*π*+1,1) and (1,1) of $\tilde{D}$ respectively to the points with coordinates given in (8.16). From figure 1, it is evident that (8.20) simply wraps, without stretching, the parallelogram $\tilde{D}$ over a cylinder of radius $1/\sqrt{2}$.

The procedure of constructing the mappings $\hat{\xi }$ and $\tilde{x}$ and a planar region $\tilde{D}$ described above can be carried out for any mapping $\hat{x}$ of the form (7.1). Alternatively, the decomposition of a non-isometric mapping $\hat{x}:D\to E^{3}$ into a non-isometric mapping $\hat{\xi }:D\to E^{2}$ and an isometric mapping $\tilde{x}:\tilde{D}\to E^{3}$ can be achieved by a change of independent variables, which effectively generates $\tilde{x}$. We briefly describe this approach in §9.

## 9. Inducing isometry by a change of variables

For a mapping $\hat{x}$ belonging to the class (7.1), we consider a change of the independent variable ***r*** defined by a mapping $\hat{\xi }:D\to E^{2}$ of the particular form

9.1 $$\hat{\xi }(r)=(r_{1}+r_{2}\eta (r_{1}))e_{1}+r_{2}e_{2}.$$

Additionally, denoting the image of D under $\hat{\xi }$ by

9.2 $$\tilde{D}=\{\xi \in E^{2}:\xi =\hat{\xi }(r),r\in D\},$$

we define a mapping $\tilde{x}:\tilde{D}\to E^{3}$ implicitly by

9.3 $$\tilde{x}(\hat{\xi }(r))=\hat{\text{x}}(r),\;r\in D.$$

Then, using the chain rule to differentiate (9.3) with respect to ***r*** and invoking (7.10), we find that the gradient ***J*** of $\tilde{x}$ on $\tilde{D}$ must obey

9.4 $$J^{⊤}J=I,$$

from which it follows that $\tilde{x}$ defined by (9.3) is an isometric mapping of the planar region $\tilde{D}$ to the surface S determined by $\hat{\text{ x}}$. It is, however, essential to recognize that the shape of the domain $\tilde{D}$ of $\tilde{x}$ is unknown unless the ratio *η*=*τ*/*κ* involving the curvature *κ* and torsion *τ* of the midline C of S is itself known. Moreover, that shape generally differs from D unless *τ* vanishes identically. The discussion of helical ribbons in §8 provides an example of this difference in the particularly simple case where *η* is constant. Any strategy that seeks to determine a mapping $\hat{\text{ x}}$ belonging to the class (7.1), say by minimizing the energy (2.2) of Sadowsky [1,2] or its generalization due to Wunderlich [7,8], will therefore generally yield a developable surface S that is isometric to a planar region $\tilde{D}$ different from the domain D of $\hat{\text{ x}}$, leaving unsolved the problem of finding the shape of an unstretchable sheet identified with a planar region D.

## 10. Alternative strategies

We have shown that the class of mappings of the form (7.1), which has been used extensively in the literature to model bands and ribbons, is not suitable when the bands and ribbons are made of unstretchable material sheets. We now offer three possible strategies for avoiding the drawbacks of working with such mappings. One of these strategies involves relinquishing the constraint of unstretchability. The other two mimic approaches that are familiar from treatments of internally constrained three-dimensional bodies.

### (a) Removing the constraint of unstretchability

Since mappings of the form (7.1) are not generally isometric and are hence inadequate for the purpose of modelling pure bendings of unstretchable flat material sheets, one possible remedy would involve dropping the isometry requirement in favour of considering stretchable flat material sheets. Among other things, this would require a modification of the elastic energy function to include the change of elastic energy induced by in-plane stretching. The Sadowsky and Wunderlich functionals (2.2) and (2.10) incorporate bending only. As a consequence, these functionals are insensitive to the energy that is required to deform, for example, the rectangular strip D in figure 1 to the parallelogram $\tilde{D}$. This is physically unreasonable. In a theory that incorporates the effect of in-plane stretching, the energy density generally depends on the stretch tensor ***U*** (or, if the flat material sheet is assumed to be isotropic, the principal stretches of ***U***), in addition to the curvature of the surface. Associated models are more complicated, both kinematically and in regard to constitutive relations. There is, however, another more fundamental and seemingly inescapable problem with this strategy. The class of mappings of the form (7.1) has been used previously because of the belief that it describes the deformations of unstretchable flat material sheets. As we have demonstrated that this belief is unfounded, a justification would therefore be needed to support continued use of such mappings in a context where stretching energy is properly incorporated.

### (b) Using strictly isometric mappings

Another possible strategy would be to replace (7.1) with the correct and complete class of isometric parametrizations. This strategy is consistent with the spirit of the literature concerning Möbius bands made from unstretchable flat material sheets, as the constraint (5.9) serves as a good approximation for a large class of two-dimensional materials, including those often used to construct model Möbius bands. It has the obvious advantage of leading to a description of great simplicity, in which the energy function depends on the mean curvature only. The task of characterizing the class of three-times continuously differentiable isometric mappings was recently addressed by the present authors and Fosdick [27]. That class neither contains nor is contained in the class (7.1), albeit there exists an intersection consisting of precisely those mappings in (7.1) with zero torsion *τ*, namely the degenerate case where the midline is planar and the conditions needed to describe a Möbius band cannot be met. Moreover, in contrast to the position of a point on a surface S determined by a mapping of the form (7.1), the position of a point on the surface mapped isometrically from a planar region R depends on the coordinates of the planar region through certain intermediate coordinates which are generated by the characteristic curves of an ordinary-differential equation associated with (6.1), which constitutes a system of the first-order differential equations for the mapping $\tilde{x}$.

### (c) Using a theory with properly imposed constraints

The third strategy, which is perhaps more familiar to workers in mechanics and which we are currently pursuing independent of the work reported here, would be to develop a theory for internally constrained flat material sheets. In such a theory, unstretchability is treated as a constraint on the class of admissible mappings used to parametrize surfaces. The theory can be developed on general grounds for a class of constraints that includes the constraint (5.9) of unstretchability as a special case. Having derived the general theory, an energy functional that incorporates bending only, and is therefore compatible with the constraint of unstretchability, can be used. The partial-differential equations of equilibrium and the complete set of edge conditions can be derived by computing the first variation of that energy functional subject to the constraint (5.9). The resulting boundary-value problem, which includes reactive forces due to the constraint, needs to be solved in conjunction with the constraint. By contrast, the approach described in the previous subsection amounts to satisfying the constraint *a priori* and substituting the result in the objective functional.

## Data accessibility

The research reported here is purely analytical and thus does not have any associated experimental data.
